# Let There Be Light for Photoproximity Labeling

**DOI:** 10.1021/acscentsci.5c01587

**Published:** 2025-09-03

**Authors:** Tae Young Han, Hyun-Woo Rhee

**Affiliations:** † Department of Chemistry, 26725 Seoul National University, Seoul 08826, Korea; ‡ School of Biological Sciences, Seoul National University, Seoul 08826, Korea

## Abstract

Bioluminescence-assisted photoproximity labeling enables spatial proteome mapping in deep tissues.

## Graphical abstract

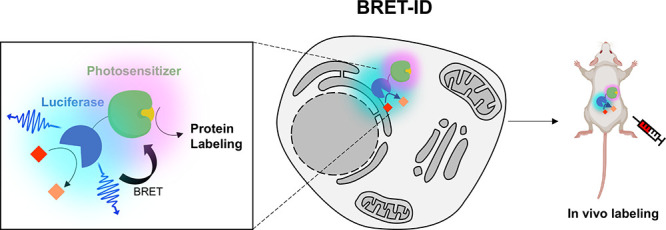

Proximity labeling (PL) has revolutionized the field of spatial proteomics. This technique generates a labile, reactive intermediate from a specific probe, which then covalently labels proximal proteins. The short half-life of this intermediate prevents its diffusion, ensuring that it reacts only with proteins in the immediate vicinity or is constrained by cellular structures such as lipid membranes. PL tools were originally developed by engineering enzymes (BirA, APX) that can catalytically generate reactive intermediates *in situ* in live cells.^1^ Enzymatic PL methods are robust; however, they require incubation time for probe penetration or enzyme activation (e.g., H_2_O_2_ for APEX2). In photocatalytic PL, “light” can be used to activate a photosensitizer or a photocatalyst, which can be a genetically encoded enzyme (e.g., MiniSOG),^2^ a heavy-metal-based catalyst (e.g., ruthenium- or iridium-based),^3^ or an organic dye (e.g., Eosin Y).^4^ After flash activation by light, photosensitizers subsequently relax to their ground state by releasing energy via an energy or electron transfer pathway. In the presence of diazirine or arylazide probes, this process can generate highly reactive intermediates, carbenes or nitrenes, respectively. Alternatively, some photosensitizers can generate reactive oxygen species (ROS) or aromatic radicals that oxidize nearby amino acid residues, rendering them susceptible to conjugation to other probes.

Beyond convenient catalyst activation, various photocatalytic reactions can be used for photo-PL that can map diverse spatial biomolecular components or the spatiome.^1^ For example, oxidized guanine, produced through a ROS-mediated photoproximity reaction, can label proximal RNAs, thereby extending the utility of this approach to the field of spatial transcriptomics.^2^ Carbene/nitrene generating photo-PL has also demonstrated powerful modifying coverage.^4^ However, existing photocatalytic PL methods are limited by nonspecific labeling resulting from unintended, endogenous photosensitizer activation during whole-cell illumination (Figure 1A). Additionally, the application of photocatalytic PL to deep tissues remains challenging due to the limited light penetration depth (Figure 1B).

**Figure 1:**
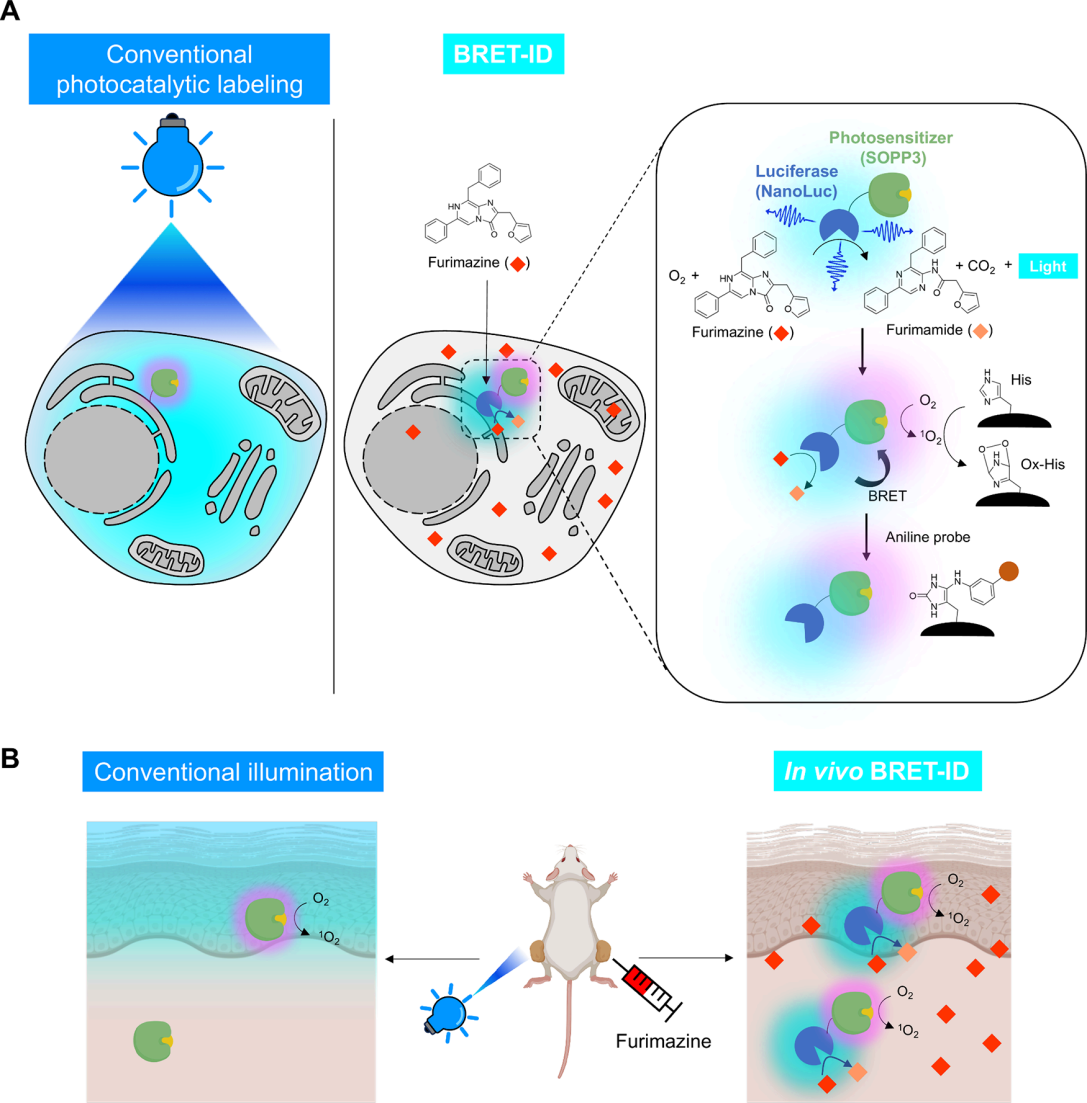
(A) Schematic overview of the BRET-ID mechanism in comparison to conventional photocatalytic proximity labeling. (B) Comparison of *in vivo* labeling scopes: conventional photocatalytic labeling vs BRET-ID.

In this issue of *ACS Central Science*, Qin and colleagues overcome the limitations of current photocatalytic PL methods by harnessing “bioluminescent” light for photoproximity labeling.

Bioluminescence arises from the chemical activation of luminophores (e.g., luciferin or furimazine) at the active site of enzymes like luciferase (e.g., NanoLuc). The emitted light is sufficiently intense to excite nearby fluorophores or quantum dots through Bioluminescence Resonance Energy Transfer (BRET).^6a^ This strategy has also been extended to activate photosensitizers, such as MiniSOG, enabling the targeted elimination of deep tissue tumors.^6b^

In the study of Qin and colleagues,^5^ the authors engineered a chimeric protein, termed BRET-ID, which fuses NanoLuc, a small luciferase from *Oplophorus gracilirostris*, and SOPP3, a member of the Light-Oxygen-Voltage (LOV) family. Upon addition, furimazine undergoes enzymatic oxidative decarboxylation at the active site of NanoLuc and emits luminescence at 460 nm. This luminescence, in turn, activates SOPP3. The activated SOPP3 then generates singlet oxygen, which oxidizes proximal proteins. These oxidized proteins are subsequently labeled by an aniline probe. Notably, 1 min treatment with furimazine was sufficient to achieve effective protein labeling.

BRET-ID’s 1 min labeling window is well suited for capturing snapshots of rapidly changing proteomes, such as the G protein coupled receptor (GPCR) interactome and stress granule proteome. BRET-ID minimally perturbs cellular proteome dynamics, as it avoids the use of high-energy light illumination or cytotoxic H_2_O_2_. Leveraging these advantages, BRET-ID successfully captured proteome dynamics over various time courses and, importantly, revealed novel interactors for both hMOR (a GPCR) and G3BP1 (a stress granule protein).

A major goal in the field of proximity labeling is to map spatial biomolecular components or the spatiome *in vivo*.

Although several studies have utilized TurboID and APEX2 *in vivo*,^1^ these approaches face limitations: TurboID suffers from high endogenous biotin labeling background, while APEX requires *ex vivo* labeling,^7^ since the H_2_O_2_ needed for its activity is too toxic to be applied directly in living organisms. While some photocatalytic PL studies have employed red light,^8^ which penetrates tissue more deeply than blue light, reaching deep tissues within a living organism such as mice remains a significant challenge. As BRET-ID only requires the administration of furimazine and a probe, the authors tested its capacity for *in vivo* labeling by injecting substrates into mice with tumor xenografts expressing G3BP1-BRET-ID (Figure 1B). Luminescence was observed only upon furimazine administration, and successful protein labeling was subsequently detected. Significantly, the G3BP1 interactome in tumor xenografts was distinct from that in cultured cells, highlighting the strength of BRET-ID for studying the spatiome in a native *in vivo* context.

We believe that BRET-ID can be further developed along several directions. For example, generating BRET-ID transgenic models (e.g., mice, zebrafish, flies) under Cre- or Gal4-dependent promoters could ensure stable expression of BRET-ID constructs in specific cell types, enabling the mapping of diverse spatiomes *in vivo*.

In addition, exploring novel combinations of bioluminescence sources^9^ with a variety of photosensitizers offers a promising avenue for creating new BRET-ID-like approaches. Within this framework, furimazine and other tissue-permeable luminophores could be tested in deep tissues and organs. The BRET-ID system could also be redesigned to map contact-dependent spatiomes when its bioluminescent and photocatalytic components are expressed separately. Finally, the spatial maps generated by BRET-ID can be compared with other *in situ* PL methods, such as d-amino acid oxidase (DAAO)-mediated production of H_2_O_2_ to activate APEX2.^10^ Since DAAO labeling and BRET-ID rely on distinct mechanisms, their comparative and combinatorial application *in vivo* could yield a more comprehensive understanding of molecular environments, which would be especially valuable for *in vivo* studies.
